# Supplementary material for: Are carbon emissions trading and green financial instruments synergistic? -Comprehensive quantitative research based on content analysis
Source: PLoS One. 2024 Mar 7;19(3):e0298601. doi: 10.1371/journal.pone.0298601 (PMC10919601; doi:10.1371/journal.pone.0298601)
Supplement: S1 Data — (ZIP) [file pone.0298601.s001.zip › Data/地级市Z_F工作报告.zip!/╔┬╬≈/╔╠┬σ╩╨/└┤╫╘╣1⁄2╓┌║┼í╛┬φ┐╦╩2╛▌═°í┐_2019.docx]

各位代表：

现在，我代表市政府向大会报告工作，请予审议，并请市政协委员和列席人员提出意见。

一、2018年工作回顾

2018年，是全面贯彻党的十九大精神开局之年，也是商洛追赶超越进程中不平凡的一年。面对各种风险挑战和繁重发展任务，全市上下坚持以习近平新时代中国特色社会主义思想为指导，坚决贯彻党中央、国务院决策部署，紧扣追赶超越和“五个扎实”要求，大力发展“三个经济”，沉着应对挑战，奋力克难攻坚，取得了好于预期的成绩。

——追赶超越迈出坚实步伐。实现生产总值824.77亿元，增长8%；规模以上工业增加值增长14.6%，居全省第二位；固定资产投资增长12.6%，民间投资增长31%；城乡居民收入分别增长8.4%和9.5%，居全省第二和第一位；财政收入质量大幅提升，税收占比提高到67.62%，地方财政收入同口径增长6.39%。180个市级重大项目完成投资544亿元。全省“十三五”重点电源建设项目商洛电厂投产运行，结束了我市没有优质可靠电源的历史。全省首条“省市共建”的山柞高速公路建成通车，开辟了区域协同发展的新通道。全省“十三五”首个采取PPP模式运作的丹凤通用机场项目投入运行，填补了我市航空产业空白。五洲高端钒系列产品开发、海恩动力电池正极材料等50个项目建成投产，镇安抽水蓄能电站、必康中药材战略储备库等项目进展顺利。150个市级重大项目前期工作扎实有效，西十、西康高铁进入可研审批程序，丹宁高速东段初步设计完成，丹宁高速西段及榆商、洛卢高速前期工作按计划推进，镇安钨钼产业园、商南镁橄榄石综合利用等69个项目具备开工条件。围绕数字经济、现代物流、军民融合、载能工业等策划项目135个，累计储备项目442个，总投资超过3000亿元。

——三大攻坚战取得丰硕成果。聚力打好脱贫攻坚战，“三带一创”产业精准扶贫模式不断深化，菌果药畜“4+X”特色扶贫产业体系基本形成，集体经济贫困村全覆盖，贫困劳动力转移就业4.46万人。贫困村基础设施得到加强，实施饮水巩固提升75万人，完成建制村畅通、“油返砂”整治和深度贫困村通组路2253.4公里，土地增减挂钩指标交易收益2.6亿元，易地扶贫搬迁和危房改造年度任务全面完成，创建“双示范”社区26个，教育、健康、生态和社保兜底扶贫成效显著。宁商扶贫协作全方位推进，争取协作资金3.16亿元，高层交流互访、项目资金援助、干部人才培训等走在全省前列。“三大帮扶”和“万企帮万村”成果丰硕，“户分三类”“四扶五风六化”等做法得到中省肯定。全市21.34万人脱贫，389个贫困村退出，镇安有望率先摘帽。全力打好青山、碧水、蓝天、净土保卫战，以坚决的态度彻底整治秦岭地区违规建筑和生态环境突出问题，达到了预期效果。中省环保督察、“绿盾行动”等反馈问题扎实整改，洛南黄龙河污染得到综合治理，二龙山水库水源地34个环境问题如期销号，中央环保督察“回头看”135个信访件按时办结。累计整治“散乱污”企业411户，植树造林34.5万亩，治理水土流失702平方公里，划定畜禽禁养区2015平方公里，整治“大棚房”问题16起，新增高标准农田4.16万亩、高效节水灌溉面积3.31万亩，市县区污水垃圾处理厂（场）提标改造和35个镇149个村环境综合整治全面完成，创建美丽宜居示范村67个，市区空气质量优良天数331天，持续位居全省第一，主要河流监测断面和城市集中饮用水源地水质稳定达标，污染减排完成省考任务。打好防范化解重大风险攻坚战，守住不发生系统性风险底线，严格管控并积极偿还政府债务8.37亿元，严厉打击非法集资、金融诈骗等违法行为，全市金融秩序总体稳定。

——高质量发展实现新突破。创新型商洛建设步伐加快，发明专利申请775件，认定高新技术企业15家，创省级以上名牌产品9个。商洛国家农业科技园区获得授牌，商洛国家级高新区列入科技部预考察计划，山阳省级高新区获批晋级，柞水入选国家首批创新型县建设名单。现代工业推进有力，重点工业项目完成投资102亿元，商南国漆产业园漆蜡生产、山阳电子信息产业园一期等18个项目建成投产，工业技改荣获全省三等奖。园区建设提档升级，新建标准化厂房20.5万平方米，新入园企业53户，洛南陶岭等5个县域工业集中区被列为省级示范园区。特色农业提质增效，划定粮食生产功能区155万亩，粮食生产实现“五连丰”，首届“农民丰收节”成功举办，国家农产品质量安全市提升示范行动率先启动，认定“三品一标”农产品29个，创建生态种养示范场54个，下梁镇入选全国农业产业强镇，达仁镇、西川村分获全国“一村一品”示范镇村，太子坪村入选中国美丽休闲乡村。全域旅游示范创建捷报频传，九天山、音乐小镇成功晋升4A级景区，金丝峡、木王山入列省级旅游度假区，商州北宽坪镇、丹凤保定村等3镇6村荣膺省级旅游特色名镇和乡村旅游示范村，全年接待旅客5851万人次。现代服务业加快发展，七县区全部跻身国家电子商务进农村综合示范县，市供销电商仓储物流配送中心、洛南西北核桃交易中心等项目建成投用，社会消费品零售总额增长11%。

——“四城联创”彰显城市新形象。中心城市创建国家卫生城市通过省级考核鉴定，国家森林城市、省级文明城市创建有序推进，省级生态园林城市创建完成，街景绿化、美化、亮化全面提升，城市绿地面积达到930万平方米，累计实施城周绿化2.6万亩，环市区及沪陕高速西段沿线直观坡面基本实现应绿尽绿，被授予省级绿化模范城市。中心城区和各县县城总体规划修编顺利推进，主城区城市设计基本完成，控制性详规实现全覆盖。城市管理体制改革不断深入，中心城市智慧城管项目基本建成。工农路改造树立了城市建设新样板，北新街东延、丹江构峪大桥路通灯亮，北郊输变电、南门停车场和东新路、东环路农贸市场建成投用，团结路、文卫路北段改造基本完工，万达广场、国际医学中心主体建成，碧桂园高端住宅项目加快推进，主城区供热管网、“气化商洛”有序实施。全市棚户区改造开工7019套，中心城区16个棚改项目梯次推进，人防违法项目清零成效明显。县城扩容提质加快，小岭梁、黄沙岭隧道顺利贯通，商南西街改造、山阳翠屏商业街等特色街区和城市综合体项目进展顺利，重点示范镇、文旅名镇和特色小镇建设走在全省前列。

——改革开放焕发出新活力。扎实开展优化提升营商环境十大行动，持续深化“放管服”改革，累计合并、取消和下放行政审批事项195项、精简率47.2%，政务服务和便民服务日益便捷高效，开办企业、获得电力、不动产登记等工作全省领先，全年为企业减税降费13.82亿元，新增市场主体1.52万户，非公有制经济占GDP比重达到56.2%，位居全省第三。农村各项改革不断深入，土地确权登记数据通过国家审验，县区农村产权交易中心建成运营，“三变”改革试点扎实推进，国有林场改革顺利完成。对外开放招商引资成果丰硕，商洛海关正式获批，森弗韩国子公司挂牌成立，华为秦岭云计算大数据中心、京东云仓等500强企业项目签约落地，招商引资到位资金增长9.11%，其中省际项目到位资金增长14.14%，利用外资增长30%。外贸进出口总额21.29亿元，位居全省第四。

——民生福祉得到持续增进。民生支出202.1亿元，占财政总支出的83.2%。城镇新增就业1.95万人，农村劳动力转移就业46.6万人。企业退休人员基本养老金、城乡居民基础养老金和医保补助标准持续提高，低保对象实现应保尽保。义务教育优质均衡发展全面推进，中小学、幼儿园建设持续加强，新增公办学位1.85万个，“大班额”问题得到缓解。医药卫生体制改革深入推进，县域内就诊率91.77%，市中心医院创三甲通过现场评审，商洛荣获全省分级诊疗先进市。文化惠民工程深入实施，建成基层综合文化服务中心355个，《商於古道》在央视播出，《情怀》获全国展演优秀剧目奖。全民健身活动广泛开展，中国环秦岭公路自行车赛等十大精品赛事成功举办。庆祝改革开放40周年系列活动丰富多彩，“七五”普法依法治理深入推进，信访工作得到加强，安全生产和食品药品监管力度加大，扫黑除恶专项斗争扎实有效，人民群众获得感幸福感安全感不断增强！

一年来，各级政府遵守宪法、依法履职，自觉接受人大、政协和纪委监委监督，主动接受审计、舆论和社会监督，市人大代表建议和政协委员提案办复率100%，网民留言和群众来信得到及时办理回复。严格落实中央八项规定及其实施细则，深化“两学一做”学习教育，认真抓好“以案促改”，扎实开展“讲政治、敢担当、改作风”专题教育，持续推进党风廉政建设，坚决遏制“四风”反弹，政府公务人员政治意识、担当精神和服务能力有效提升。与此同时，国防动员、民族宗教、外事侨务、防震减灾、决策咨询、统计、方志、气象、邮政、档案、老龄、无线电，以及妇女儿童、慈善、红十字、残疾人和关心下一代等工作都取得了新成绩。

各位代表！奋斗历经艰辛，成绩来之不易。在脱贫攻坚主战场，广大基层干部、第一书记、驻村队员，用责任、奉献和忘我的付出，温暖和鼓舞着每一名贫困群众；在秦岭地区违规建筑和生态环境突出问题整治中，各级各部门坚决贯彻习近平总书记重要批示指示，用实际行动和成效诠释“两个维护”的政治担当；在各类灾害和突发事件处置现场，广大党员干部不畏艰险、冲锋在前，极力维护人民群众生命财产安全。这些可歌可泣的感人事迹，凝聚着加快发展、追赶超越的正能量，激发着拼搏进取、砥砺前行的原动力。在此，我代表市政府，向全市广大干部群众、驻商部队、公安干警、消防指战员致以崇高敬意！向关心支持政府工作的各位代表、各位委员和社会各界人士表示衷心感谢！

二、2019年思路目标

2019年是新中国成立70周年，是全面建成小康社会关键之年，也是我市脱贫攻坚决战决胜之年。

我们深知，前进道路上困难和挑战不容忽视。受宏观形势影响，需求增速放缓，经济下行压力加大，加之我市发展基础比较薄弱，追赶超越支撑点和突破点不多；企业规模普遍偏小，产业链条较短，科技含量不高；县域经济实力不强，七县区在全省和陕南排名整体靠后；财政收入增长乏力，收支矛盾突出；生态保护、脱贫攻坚任务十分艰巨，民生领域还有不少短板；形式主义、官僚主义不同程度存在等等。

同时，我们也面临诸多机遇和利好。中央继续实施积极的财政政策和稳健的货币政策，强化竞争政策的基础性地位和社会政策的兜底保障功能，大力实施“一带一路”、乡村振兴等重大战略，倾斜支持深度贫困地区和关中平原城市群建设。我省大力发展“三个经济”，协调推进陕南绿色循环发展和西商融合互补发展。近年来，我们着力强基础、补短板、调结构、兴产业，一批重大项目相继建成并陆续发挥效益，这些都将为加快追赶超越、推动高质量发展注入新活力、增添新动能。只要我们保持战略定力，坚定必胜信心，瞄准目标精准施策，抓住机遇迎难而上，就一定能够化危为机、破浪前行，向着全面建成小康社会目标，迈出更加坚实的步伐！

今年政府工作的指导思想是：以习近平新时代中国特色社会主义思想为指导，全面贯彻党的十九大和十九届二中、三中全会精神，统筹推进“五位一体”总体布局，协调推进“四个全面”战略布局，坚持稳中求进工作总基调，坚持新发展理念，坚持推动高质量发展，以供给侧结构性改革为主线，以深化改革、扩大开放为动力，以脱贫攻坚为统揽，聚焦追赶超越和“五个扎实”，紧扣“六个稳”要求，全面落实“五新”战略任务，继续打好“三大攻坚战”，大力发展“三个经济”，持续推进“五个战役”，统筹做好稳增长、促改革、调结构、惠民生、防风险各项工作，保持经济持续健康发展和社会大局稳定，以优异成绩庆祝中华人民共和国成立70周年。

主要预期目标是：生产总值增长7.5%-8%，规模以上工业增加值增长12%，全社会固定资产投资增长9%，社会消费品零售总额增长10%，财政总收入和地方财政收入同口径分别增长6.5%、8%，城乡居民收入分别增长8%和8.5%，城镇登记失业率、居民消费价格指数涨幅分别控制在4.2%和3%以内，生态环保和节能减排指标完成省上下达任务。

提出上述目标，既落实稳中求进和脱贫摘帽要求，又体现追赶超越和高质量发展目标。实现上述目标，必须全面贯彻“巩固、增强、提升、畅通”八字方针，着力打造供给侧结构性改革“升级版”。巩固“三去一降一补”成果，推动更多产能过剩行业加快出清，降低全社会各类营商成本，加大基础设施等领域补短板力度。增强微观主体活力，发挥企业和企业家主观能动性，着力破除各类要素流动壁垒，建立公平开放透明的市场规则和法治化营商环境。提升产业链水平，注重利用技术创新和规模效应形成新的竞争优势，推进产业链、创新链、资金链、政策链有机融合，打造特色现代产业集群。畅通国民经济循环，加快建设统一开放、竞争有序的现代市场体系，提高金融体系服务实体经济能力，形成国内市场和生产主体、经济增长和就业扩大、金融和实体经济良性循环。

三、2019年重点任务

（一）一鼓作气打赢脱贫攻坚战，筑牢全面建成小康社会的坚实基础。瞄准目标、聚力攻坚，切实解决“两不愁三保障”突出问题，确保10.52万贫困人口脱贫，259个贫困村退出，县区全部摘帽。

狠抓产业就业扶贫。加大产业扶贫投入，持续深化“三带一创”，大力推行“1+1>2”的产业脱贫模式，培育“4+X”特色产业体系，继续抓好核桃、板栗、食用菌、中药材、茶叶等产业发展，完善带贫益贫机制，壮大村集体经济，促进2.46万户8.03万人稳定增收。搞好贫困人口就业技能培训，加强就业扶贫基地和社区工厂等载体建设，吸纳更多贫困劳动力就近就地就业。

改善基础设施条件。实施农村饮水安全巩固提升20万人，完成深度贫困村通组公路471.7公里、通村公路“油返砂”整治598.8公里。全面完成易地扶贫搬迁安置房及各项配套建设任务，切实提高实际入住率。扎实推进易地搬迁户旧房腾退、宅基地复垦，完成建档立卡贫困户危房改造扫尾工程350户。强化移民社区服务管理，创建市级“双示范”社区示范点100个。

凝聚脱贫攻坚合力。持续做好生态、教育、健康、社会保障等扶贫工作。巩固扩大宁商扶贫协作成效，继续搞好人才支援、劳务协作、“携手奔小康”等重点工作，推进国企、高校、卫计对口帮扶和“万企帮万村”行动。大力实施消费扶贫，提升“爱心超市”管理水平，发挥中国社会扶贫网平台作用，广泛动员社会力量参与脱贫。

构建脱贫长效机制。落实脱贫不脱政策等要求，保持帮扶力量、扶持政策的连续性和协调性，研究解决收入略高于建档立卡贫困户群体缺乏政策支持等问题，提高脱贫质量和实效。坚持智志双扶，整合镇村综合文化站、农家书屋等教育阵地，建设新时代文明实践中心，深化“四扶五风六化”精神扶贫，大力宣传表彰一批先进典型，凝聚合力攻坚正能量，倡导脱贫光荣新风尚。

（二）深入实施乡村振兴战略，扎实推进农业农村现代化。坚持农业农村优先发展，持续深化农业供给侧结构性改革，大力发展特色现代农业，提高农业农村发展质量和水平。

夯实农业发展基础。加快编制村庄规划，坚持资源规划多规合一，实现主体功能区规划、城乡建设规划、土地利用总体规划有效衔接。深入推进国土空间综合整治，完成建设用地增减挂钩跨省调剂节余指标3000亩，搞好第三次国土外业调查。持续实施农业综合开发，提升改造基本农田，发展高效节水灌溉，治理水土流失600平方公里，加快推进商州桃庄、洛南台峪、山阳磨沟、镇安云镇、商南清油河等水源水库建设。

发展特色现代农业。依法保护粮食生产功能区，粮食总产稳定在48万吨左右，严密防范非洲猪瘟等疫情，提高农产品有效供给。积极创建国家特色农产品优势区，新建核桃示范园7万亩，板栗嫁接改造8.5万亩，发展食用菌1.9亿袋、中药材32万亩。抓好“三品一标”认定，推进国家农产品质量安全市提升示范行动。发展新型农业经营主体，新增市级现代农业园区、龙头企业、示范合作社和家庭农场各10个，培育新型职业农民1700人，清理“空壳”合作社，打造产业化联合体，构建现代农业经营体系。推进农村“双创”，培育休闲农业、创意农业、数字农业等新型业态，促进农村一二三产融合发展。

改善农村人居环境。学习浙江“千万工程”经验，围绕垃圾污水处理、厕所革命、村容村貌提升，抓好农村人居环境整治。加快“四好农村路”建设，实施农网改造、行政村光纤全覆盖等工程，加快补齐农村基础设施和公共服务短板。大力发展乡村旅游，不断拓展农田生态保育和乡村大景观功能，支持建设一批田园综合体。

（三）下大气力抓好项目建设，着力强基础补短板增后劲。充分发挥有效投资关键作用，狠抓项目策划、落地实施和要素保障，为加快追赶超越提供强大支撑。

狠抓重大项目建设。抓好200个市级重大项目，完成年度投资420亿元。积极推动西十、西康高铁和高速公路项目建设，力争丹宁高速东段年内开工，洛卢高速完成PPP项目招商，丹宁高速西段、榆商高速完成初步设计。加快镇安抽水蓄能电站、必康中药材战略储备库、204所含能材料产业园等项目建设进度，力争森弗数字化菊芋制造中心、盘龙技改等80个项目建成运行。扎实做好通用航空开发、石墨产业园等150个重大项目前期工作，力争丹凤年产20万吨碳酸钙、商州红星美凯龙家居综合体等50个以上项目具备开工条件。

强化项目要素保障。优化项目审批服务，大力推行投资项目在线审批和并联审批，严格落实限时办结制。积极争取基础设施补短板、脱贫攻坚、民生工程等中省政策资金支持，用足用好地方政府债券资金，强化土地和基础设施等保障。健全融资担保体系，支持金融机构更好地服务实体经济，鼓励民间资本规范有序参与PPP项目，进一步激发民间有效投资活力。

加强项目策划储备。对接政策取向和资金投向，完善重大项目策划储备工作机制，积极引入第三方咨询机构，围绕脱贫攻坚、特色产业、基础设施、城市建设、生态旅游、先进制造业、现代服务业等领域，高质量策划一批重大项目、培养一批项目人才。建立政府投资三年滚动储备项目库、专项债券项目库、新兴产业项目库和服务业发展项目库，全年策划储备重大项目不少于400个。

（四）积极培育新动能，全力推动高质量发展。围绕构建现代化产业体系，改造提升传统产业，加快发展战略性新兴产业和现代服务业，着力提升产业发展质量、效益和水平。

深化创新型商洛建设。加快商洛国家级高新区创建，力争上半年通过科技部考察验收，推动山阳省级高新区和各县域工业集中区转型提质。搞好商洛国家级农业科技园区、柞水国家级创新型县建设，支持山阳、商南创建省级创新型示范试点县，推进镇安、丹凤创建省级农业科技园区。加大知识产权创造运用管理和保护力度，建成市级科技资源统筹中心，组建市级工程技术研究中心16个，实施科技成果转化项目10个，培育高新技术企业6家、科技型中小企业和瞪羚企业16家。

培育战略性新兴产业。深入实施新一轮技术改造升级工程，改造提升传统产业，落实中国制造2025商洛实施方案及重点产业推进方案，延伸补强新能源、新材料、动力电池、智能制造、生物医药、绿色食品等产业链，重点抓好2000吨动力电池负极材料、泰华医药产业园、硅科技产业园、丰源钒系列产品清洁生产等40个重点工业项目建设。落实工业高质量发展“14条”，持续开展企业帮扶、工业招商等活动，新增产值过亿元企业10户，培育规上工业企业20户。

加快全域旅游示范创建。迎接高铁时代的到来，进一步发挥生态、交通、区位等优势，突出抓好景区拓展、跨界融合、节赛营销，带动发展低空旅游、智慧旅游、休闲旅游等新业态，优化提升精品线路、精品景区和景点，支持国家旅游度假区和高A级景区创建，力争商南、山阳、柞水创建国家全域旅游示范县通过验收。完成市县区游客中心和智慧旅游平台建设，办好秦岭生态旅游节和十大品牌节庆活动，全年旅游综合收入增长15.5%。

发展壮大现代服务业。实施消费升级行动计划，加快发展现代物流、电子商务、融资租赁、众创众筹等现代服务业，积极培育健康养生、医疗保健、智慧养老等新业态，促进制造企业和生产性服务企业资源整合、业务融合，拓展市场信息、产品技术、管理咨询等生产性服务业，抓好创业大街、秦岭农产品交易中心、金山养老公寓等项目建设，推动柞水、商南聚利村镇银行开业运营。落实个人所得税专项附加扣除等政策，提高城乡居民消费能力。

（五）坚决打好污染防治攻坚战，守护好商洛青山蓝天碧水净土。扛牢生态环境保护的政治责任，坚定不移走生态优先、绿色发展之路，让商洛天更蓝、山更绿、水更清、环境更优美。

推进绿色循环发展。坚持生态产业化、产业生态化，发展生态经济，构建绿色产业体系。推动资源综合利用，搞好园区循环化改造和产业链延伸，继续抓好国家气候适应型城市重点实验室建设，“商洛·中国气候康养之都”获得授牌。加快商南莲花台等水电站建设，新增水电装机4800千瓦，做好商州闫村等风电项目前期，推进煤改气、煤改电和新能源利用。抓好农业废弃物资源化利用，推动农业绿色循环发展。

强化生态环境保护。修订完善我市秦岭生态环境保护总体规划，推进四级网格化监管体系和平台建设，下大气力巩固秦岭地区违规建筑和生态环境突出问题专项整治成果。抓好中省环保督察反馈问题和城市集中式饮用水源地环境问题整改，彻底清理整治“大棚房”。巩固“田长制”，强化永久基本农田保护，坚决守住耕地红线。推进“林长制”，持续开展保护森林资源严打整治行动，抓好松材线虫病防治，完成植树造林35万亩、退耕还林9.9万亩、封山育林40万亩，市区城周绿化补植提升5000亩。抓实“河长制”，大力整治非法采砂采石等问题，推进绿色矿山建设，每县区至少建成一个山水林田湖草综合示范项目。搞好第二次全国污染源普查和生态保护红线划定工作。加强领导干部自然资源资产离任审计，进一步夯实各级生态环境保护责任。

重拳整治污染问题。实施铁腕治霾打赢蓝天保卫战三年行动，精准抓好“散乱污”企业清洁化改造，排查取缔重污染“10+3”小企业，系统推进“减煤、控车、抑尘、治源、禁燃、增绿”措施，确保大气质量稳定向好。实施丹江等流域污染防治四年行动计划，强化河流水系整治和水源地保护，搞好二龙山水库水源地环境问题整治收尾和成果巩固，全面完成县级集中式饮用水源地环境突出问题整改，强力推进污水垃圾处理PPP项目建设，确保城市饮用水源地和主要河流水质全面达标。加强土壤污染风险管控，抓好固体废物污染专项整治，推进土壤污染治理与修复，呵护好商洛的青山绿水。

（六）推动城市扩容提质，加快新型城镇化发展步伐。完善城市规划，强化城市管理，加快县域经济发展，抓好城镇基础设施和重点镇建设，提升新型城镇化发展内涵。

提升城市规划管理水平。完成中心城市及各县县城总规修编，以及县城绿地系统控制性详规和专项规划、村镇建设规划编制工作。深化城管执法体制改革，加快县区智慧城管项目建设，提高城市人性化、便利化建设、管理和服务水平。严格规划执法，扎实开展市容环境、违法建设、污水垃圾和广告牌匾专项整治，全面推进生活垃圾分类处理。深入推进“四城联创”，确保国家卫生城市通过国家爱卫办暗访和技术评估，国家园林城市、森林城市和省级文明城市完成年度创建任务。规范房地产市场秩序，加快建立多主体供给、多渠道保障、租购并举的住房制度。加强建筑业和住宅物业管理，完成居民小区供配电设施移交改造，改善居民生产生活环境。

加大中心城市建设力度。完成文卫路提升改造和植物园、高新公园、南秦湿地公园二期等项目建设任务，推进城市综合管廊、集中供热、农贸市场、公共厕所、停车场、充电桩等设施建设，启动中心城市东门户区和市人防指挥所建设，抓好江南小区、丹南新区等重点区域绿化美化。6月底前万达广场开业运营、商州至洛南城市干线全线通车，年底前碧桂园高端住宅项目主体完工、国际医学中心投入运营。围绕西十高铁商洛西站规划建设，搞好南秦新区规划修编，加快龟山隧道、南秦大桥和南秦片区污水管网施工建设进度，启动南秦水厂、环城南线西段建设，做好高铁大道、高铁综合体等项目前期工作。

推进县域经济及城镇化发展。抓住中省支持县域经济发展和城镇建设政策机遇，实施县域经济发展三年行动计划，着力提升县域经济整体实力，推动洛南撤县设区。加快县城扩容提质，搞好丹凤江南新城、山阳翠屏山公园、柞水凤凰过境路等城镇配套设施项目建设。开展“棚改提质年”活动，推进43个续建、5个新建棚改项目，新开工2450套、建成安置房1864套。加快“气化商洛”进程，商州至洛南、丹凤、山阳长输管线供气，推进丹凤至商南长输管线建设，提高镇安、柞水县城天然气利用水平。抓好漫川关、棣花等省市重点镇建设，加快小城镇发展。

（七）全面深化改革扩大开放，激发市场主体活力。坚定不移推进供给侧结构性改革，持续优化提升营商环境，提高开放合作和招商引资水平，不断增强发展的内生动力。

深化重点领域改革。持续优化营商环境，深化“放管服”改革，推进相对集中行政许可权改革，实现“一枚印章管审批”，严格执行“三级四同”政务服务事项目录，建成全市政务信息共享平台，加快“一网通办”，网上审批率不低于70%。深化农村土地制度改革，鼓励支持土地经营权有序流转，推进农村集体产权制度改革，搞好丹凤县国家级改革试点，健全农村产权交易体系。深化国资国企改革，完成“三供一业”分离移交，推进县区经营性国有资产集中统一监管。全面完成政府机构改革，深化事业单位和综合行政执法改革，推进供销、盐业、科技、文化和安全生产等领域改革，搞好第四次全国经济普查工作。

大力发展“三个经济”。把发展枢纽经济、门户经济、流动经济，作为加快新时代追赶超越步伐的重要抓手，围绕高速、高铁、航空等现代交通，加快构建陆空互动、多式联运的综合交通枢纽体系，抓好商洛陆港等公铁联运项目和秦岭飞行小镇建设，主动跟进陕西自贸区建设，确保商洛海关下半年动工建设。加快发展数字经济，促进互联网与实体经济深度融合，抓好秦岭云计算大数据中心、京东云仓等项目建设，推动大数据、云计算、人工智能、跨境电商等产业发展，支持和引进国内外物流龙头企业来商发展，推广森弗跨境电商新模式，完成电商交易额54亿元。

激发民营企业发展活力。全面落实中省支持民营经济发展的各项政策措施，着力消除民营企业在准入许可、经营运行、招投标等方面的不公平待遇。落实更大规模的减税降费等扶持优惠政策，探索建立民营企业纾困基金和融资奖补机制，健全完善企业融资担保体系，着力解决民营企业融资难问题。支持企业拓宽融资渠道，促进陕西天之润、丰源钒业等企业上市，推动8家企业在区域股权市场挂牌。完善民营企业参与涉企政策制定机制，畅通政企沟通渠道，构建亲清政商关系。

加大招商引资力度。坚持内资与外资并重，瞄准国内外重点区域，抓住“一带一路”建设等机遇，面向我市外资主要来源国家和地区，积极探索赴境外、国外开展专题招商活动，依托丝博会、进博会及陕西—长三角、粤港澳经济合作活动等平台，开展叩门招商、组团招商，推动华为、绿地、恒大、三一重工等500强企业来商投资，全年招商引资到位资金720亿元。积极设立海外展示中心，新增外贸企业10家，抓好笃诚医药、南京外贸服饰加工产业转移等项目建设，全年外贸进出口22亿元，利用外资2200万美元。

（八）坚持以人民为中心的发展思想，持续保障改善民生。全面发展社会事业，完善社会保障体系，加强社会治理，防范化解风险，不断满足人民群众对美好生活的向往。

推进各项社会事业健康发展。统筹推进城乡义务教育一体化，继续抓好城区中小学校建设和“全面改薄”等项目，持续消除“大班额”，推动义务教育优质均衡发展，全面推行学校安全标准化管理。支持商洛学院建设，鼓励商洛职院多渠道发展，支持和规范社会力量办学，推动各类教育协调健康发展。落实“健康商洛2030”行动计划，开展健康细胞示范建设，深化公立医院综合改革，加快推进分级诊疗制度建设，推动市中心医院扩建、市中医医院升级改造等项目建设。深入开展爱国卫生运动，加强疾病预防控制，不断优化计生服务。严格依法保护文物，大力发展文化产业，打磨文艺精品，加快商於古道、市博物馆等重点项目建设，广泛开展文化惠民进基层和全民健身活动，加强十四运排球场等体育场馆和健身设施建设，高质量办好环秦岭国际公路自行车赛等十大精品赛事。

健全完善社会保障体系。把稳就业放在更加突出位置，统筹做好高校毕业生、农村转移劳动力、城镇失业人员、退役军人等重点群体就业，新增城镇就业1.6万人。深化收入分配制度改革，建立完善工资增长机制，强化工资尤其是农民工工资支付保障。继续实施全民参保计划，推动社会保障实现法定人群全覆盖。将社会救助规范化镇村创建与农村低保专项治理有机结合，全面落实兜底保障政策。健全社会救助体系，支持和发展红十字、慈善、残疾人和关心下一代事业。完善社会养老保障体系，加快15个城市社区日间照料中心和155个农村互助幸福院等项目建设。

推动平安商洛建设。坚持底线思维，扎实开展防范化解重大风险三年行动计划，加强政府性债务风险管控，推动融资平台公司市场化转型，全面实施预算绩效管理，强化财政专项资金和政府投资项目监管。加大财源建设和培育力度，持续提高收入质量，税收占比达到70%。加强社会信用体系建设，加大金融机构不良贷款处置力度，确保不发生系统性金融风险。持续开展重点领域安全攻坚行动，强化食品药品监管，健全应急管理体系，统筹做好防灾减灾救灾工作。深化“七五”普法依法治理，持续推进法治信访建设，加强人民调解工作，深入开展扫黑除恶专项斗争，加快实施“雪亮工程”，严厉打击各类违法犯罪活动，努力建设更高水平的平安商洛。

四、全面加强政府自身建设

面对当前形势和任务，各级政府必须讲政治、敢担当、改作风、善作为，切实肩负起新时代赋予的职责和使命，为全面深化改革、推动高质量发展、持续改善民生而不懈努力。

旗帜鲜明讲政治。深入贯彻习近平新时代中国特色社会主义思想和党的十九大精神，持续强化思想武装，增强“四个意识”，坚定“四个自信”，做到“两个维护”，始终在思想上政治上行动上同以习近平同志为核心的党中央保持高度一致，在重大原则问题上立场坚定，在大是大非面前旗帜鲜明，在贯彻中央决策部署上态度坚决，确保党中央令行禁止。

不辱使命勇担当。始终站在党和人民立场上，以事业为先、以大局为重、以民生为本，忠诚履职、尽职尽责。敢于冲锋陷阵，积极破解改革发展中的困难和问题。敢于坚守原则，自觉弘扬正气，抵制歪风邪气。敢于牺牲自我，努力拼搏进取，无私奉献。用好用活“三项机制”，为敢担当、善担当的人撑腰鼓劲，激励广大干部在新时代有新担当、新作为。

持之以恒转作风。持续整治“四风”，紧盯形式主义、官僚主义新动向新表现，切实解决表态多调门高、行动少落实差等问题，坚持谋在深处、干在实处，说了就办、办就办好，多干一些群众看得见、摸得着、得实惠的实事好事。规范督查检查，大力精文减会，多措并举为基层“减负降压”、为干部“松绑解套”。坚持依法行政、依法办事，善于运用法治思维和法律手段解决问题、推动落实。

毫不松懈抓廉政。认真履行全面从严治党主体责任和“一岗双责”，严格落实中央八项规定及其实施细则，持续做好“以案促改”、警示教育“后半篇文章”，深入推进政府系统党风廉政建设和反腐败斗争，既要坚决查处各类违法违纪案件，也要切实解决发生在群众身边的不正之风和腐败问题，切实做到干部清正、政府清廉、政治清明。

各位代表，成绩不是等出来、喊出来的，而是拼出来、干出来的。让我们更加紧密地团结在以习近平同志为核心的党中央周围，高举习近平新时代中国特色社会主义思想伟大旗帜，在省委、省政府和市委坚强领导下，上下同心、迎难而上，奋力谱写新时代追赶超越新篇章，以优异成绩向新中国成立70周年献礼！
